# A novel scoring system to analyze combined effect of lifestyle factors on pancreatic cancer risk: a retrospective case-control study

**DOI:** 10.1038/s41598-017-13182-w

**Published:** 2017-10-20

**Authors:** Tianshu Pang, Guoping Ding, Zhengrong Wu, Guixing Jiang, Yifei Yang, Xiaofei Zhang, Liping Cao

**Affiliations:** 1 Department of General Surgery, Sir Run Run Shaw Hospital, School of Medicine, Zhejiang University, No. 3, Qingchun Road, Hangzhou, China; 2 Department of General Surgery, First People’s Hospital of Yuhang District, Hangzhou, China; 3 Department of Clinical Epidemiology & Biostatistics, Second Affiliated Hospital, School of Medicine, Zhejiang University, No. 88, Jiefang Road, Hangzhou, China

## Abstract

Although several risk factors for the onset of pancreatic ductal adenocarcinoma (PDAC) have been identified, currently, no scoring system to systemically evaluate the risk of PDAC has been established. In this study, we aimed to use a population of over 1200 patients to build a novel scoring system, and evaluated combined effects of risk factors for PDAC patients.A set of 4904 participants including 1274 PDAC patients and 3630 non-cancer individuals were recruited for the single-center study over 17-year period (1997~2013). Systematic logical analysis were presented for case and control groups, and a risk rating system was constructed to assess combined risk factors. Seven independent risk factors were identified with the increased risk of PDAC, were selected into the risk score. A merged risk assessment model was established, demonstrating significantly increased PDAC risk in following a number of rising scores. Individuals with scores from 1 to more than 4, the responding OR (95% CI) were 3.06 (2.57~3.65), 7.08 (5.63~8.91), 22.4 (14.2~35.4), and 31.4 (12.7~77.5), respectively. The integer-based risk score in the study can be used for risk stratification to accurately evaluate PDAC occurrence at an early stage. This scoring system provides an accurate risk assessment of PDAC risk.

## Introduction

Pancreatic ductal adenocarcinoma (PDAC), the most common type of pancreatic malignancy, is a high lethal pancreatic tumor. Pancreatic cancer is the fourth leading cause of cancer death in the United States and the twelfth in the world^1–3^. At least 250,000 individuals worldwide are suffered PDAC annually, which accounts for around 3% systemic malignant tumors^4,5^. It is estimated that PDAC would become the primary cause of cancer death in 2050^4^. With regard to China, about 32,100 deaths were attributed to PDAC in 1990, and this number was rapidly increased to 58,200 in 2010 ^6^. According to statistics, 85% of patients with PDAC were diagnosed at a late stage^7^. An annual report from 1975 to 2002 indicated that the 5-year survival rate of patients involved with curative resection had reached to 20%, however, only 10~15% of all the PDAC patients had access to radical surgery, and the majority died within one or two years after diagnosis^1,8^. The overall 5-year survival rate is about 8%, being improved slightly over the past five years^3^.

The investigation of the relationship between PDAC and its pathogenesis has been increased since the increasing prevalence of pancreatic malignancy worldwide. Single risk factor assessment studies show that 30%~40% of PDAC cases are attributable to known factors including family history, genetic disorders, environmental exposure, tobacco use, occupation and job exposures, medical conditions and lifestyle factors^5^. Both demographics and medical characteristics have an effect on the increased risk for PDAC occurrence, such as smoking^5,9,10^, heavy amounts of alcohol drinking^11–14^, diabetes^15–18^, chronic pancreatitis^19,20^, and family history of PDAC^5,21^. However, information on epidemiologic characteristics of multiple risk factors associated with PDAC is very limited^22^. To evaluate the effect of multiple risk factors on PDAC developing, we scored Chinese participants based on their conformity to risk rating using certain potentially modifiable rik factors. Associations between combined variables multiple risks of PDAC were examined.

## Materials and Methods

### Study design

**Figure 1:**
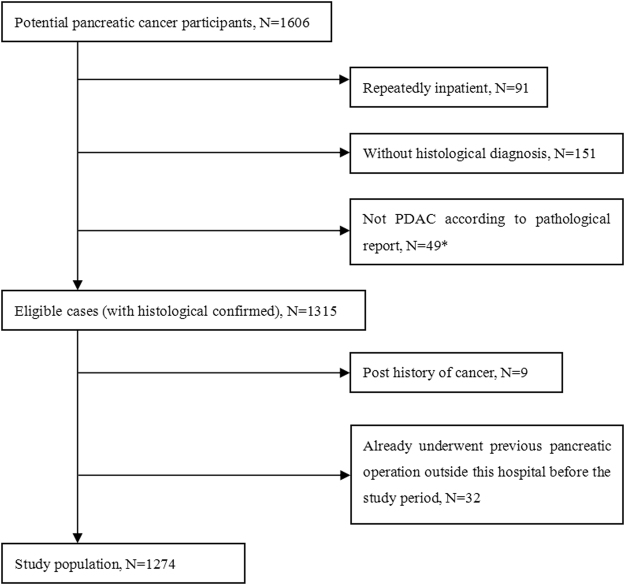
Screening of flow chart for PDAC patients.

This was a retrospective case-control study. Records from patients undergoing PDAC from 1st July 1997 to 30th June 2013 from clinical database of our medical centers in affiliated hospital of Zhejiang University medical college, were evaluated to assess risk factors for PDAC development. Stepwise screening involved 1274 eligible patients (805 males/469 females) (Fig. 1) and 3630 health individuals (1835 males/1795 females) were selected for case group and control group. Since PDAC patients were among 41~93 years old, we excluded those younger than 40 and older than 90 years from control group. The controls were ordinarily residents in the same area, going through annual routine physical examination in the same hospital between 1997~2013. The participants did not have a family history of pancreatic cancer nor history of malignant tumors. Patients underwent surgical procedure, including radical, palliative or exploratory operation were diagnosed by postoperative histopathological examination, and others without surgery were diagnosed by preoperative fine needle aspiration pathology. Both case and control groups were local Chinese residents from Zhejiang, Anhui, Jiangsu and Jiangxi Province.

### Data collection

The research protocol was reviewed and approved by the Research Ethics Committee of Sir Run Run Shaw Hospital, School of Medicine, Zhejiang University. The Institutional Review Board of the hospital approved the query of a maintained database to identify inpatients with the diagnosis of pancreatic cancer. The local ethics committee of the hospital also approved the study protocol. A dedicated interview was cond ucted face to face with each patient by trained doctors that equipped with qualification certificate. And physicians of health examination recorded basic medical information of the controls. We got access to medical records of case group from Hospital Record Department. The protocol in this retrospective clinical study was performed in accordance with the principles of Helsinki Declaration.

The information collected includes general situation of age, sex, weight, height, smoking and drinking habits, family history of diseases, medical history of gastritis, chronic pancreatitis (CP), cholelithiasis, cholecystectomy, gallbladder polyps, hypertension, diabetes (DM). The preoperative blood specimen collection was taken following a standardized checklist early next morning before breakfast in hospitalization. The preoperative blood specimen collection includes fasting blood glucose (FBG), albumin/globulin (A/G) ratio, alanine transaminase (ALT), aspartate aminotransferase (AST), alkaline phosphatase (ALP), gamma-glutamyl transpeptidase (GGT), alpha-L-fucosidase (AFU), total cholesterol, triglycerides, high-density lipoprotein-cholesterol (HDL-C) and low-density lipoprotein-cholesterol (LDL-C). Smokers/drinkers were defined as those who had been continuously or cumulatively smoking/drinking for at least one year in their lifetime, whereas nonsmokers/nondrinkers were defined as those who had not^23^. The amount of smoking was expressed by pack-years, which is equal to number of cigarettes smoked per day × number of years smoked/20 (1 pack has 20 cigarettes)^24^. Alcohol consumption was classified as low dose (0~1 drink/day) and high dose (>1 drink/day)^25^. Family history of PDAC was collected among first-degree relatives, and those with familial cancer syndromes were excluded. TC/HDL-C ratio is calculated with a demarcation point of 5 ^26^. Among case and control groups, people with gallstones or gallbladder polyps had been ultrasonic confirmed; people with chronic gastritis had gastroscopy report accordingly; people with chronic pancreatitis were diagnosed in accordance with the guidelines^27^. Participants of diabetes all belonged to the type II diabetes, which were categorized according to the American Diabetes Association diagnostic criteria^28^. The duration of the above diseases was diagnosed at least 12months.

### Statistical analysis

Univariate analysis was performed for each variable, using Pearson’s chi-square test for categorical variable and nonparametric Mann–Whitney U test for continuous variable. Unconditional univariate and multivariate logistic regression analyses were used to estimate odds ratio (OR) and corresponding 95% confidence interval (95% CI) in order to quantify the risk associated with PDAC development. All factors with a P value of <0.05 and with potential clinical relevance were analyzed into a multivariate logistic regression analysis. Related variables were put through forward and backward stepwise regression of likelihood ratio in unconditional binary logistic regression analysis. To further investigate the influence in combined risk predictors, we performed a risk rating system to determine associations with PDAC occurrence. The referent for each independent variable was assigned a value of 0. Individual score was assigned to each participant’s record by summing the score of risk factor points. Validity was assessed by the same method for each record. Statistical analyses were carried out using PASW 20.0 software (SPSS, Chicago, IL, USA). P values were based on two-sided tests and values of P < 0.05 were considered statistically significant for all statistical analyses.

## Results

### The basic information of studied objects

In our study, 88.2% PDAC patients had their duration of the disease less than 6 months, 64.1% had their location in pancreas head to neck, and 26.2% underwent radical surgery. As shown in Table 1, the average age of patients was 63.5 ± 11.3 years old, which of control group was 63.1 ± 6.6 years old, indicating that individuals in two groups were comparable (P > 0.05). Males accounted for 63.2% of the components of pancreatic maligent tumors. Men might be responsible for a positive risk factor. Compared with the control group, the percentage of PDAC patients was higher in smoking, family history of PDAC, diabetes, chronic pancreatitis, and cholecystectomy. A lower proportion of case group compared with the control group was seen in gastritis and gallbladder polyps. Blood indicators also demonstrated statistic differences of abnormal liver function and dyslipidemia. There were a higher TC/HDL-C ratio and a lower BMI in the case group, compared to the control group.

**Table 1:** Demographics and Clinical Characteristics in cancer patients and healthy subjects.

Variable	Cases (n = 1274)	Controls (n = 3630)	Wald χ2/Z	P
Male gender	805 (63.2)	1835 (50.6)	60.6	<0.001
Age (years)	63.5 ± 11.3	63.1 ± 6.6		0.169
BMI (kg/m^2^)	20.7 (18.7–22.7)	24.4 (22.4–26.5)	−32.1	<0.001
Smoking	529 (41.5)	998 (27.5)	86.57	<0.001
Alcohol Drinking	439 (34.5)	1246 (34.3)	0.007	0.931
Family History of PDAC	35 (2.7)	48 (1.3)	11.51	0.001
Diabetes	203 (15.9)	297 (8.2)	61.9	<0.001
Gastritis	26 (2.0)	88 (2.4)	0.61	0.435
Pancreatitis	53 (4.2)	6 (0.2)	126.6	<0.001
Cholelithiasis	123 (9.7)	307 (8.5)	1.69	0.194
Prior History of Cholecystectomy	64 (5.0)	26 (0.7)	97.12	<0.001
Gallbladder polyps	28 (2.2)	360 (9.9)	77.13	<0.001
FBG (mmol/L)	5.77 (5.11–6.83)	5.11 (4.78–5.56)	−17.7	<0.001
TC (mmol/L)	4.27 (3.49–5.03)	4.99 (4.28–5.65)	−14.7	<0.001
Triglycerides (mmol/L)	1.30 (0.94–1.83)	1.40 (0.96–2.08)	−2.58	0.01
HDL-C (mmol/L)	1.11 (0.80–1.42)	1.44 (1.19–1.71)	−18.3	<0.001
LDL-C (mmol/L)	2.35 (1.76–3.0)	2.94 (2.33–3.47)	−12.8	<0.001
TC/HDL-C ratio	3.59 (2.94–5.22)	3.41 (2.88–4.06)	−7.88	<0.001
Albumin/Globulin	1.57 (1.35–1.79)	1.75 (1.58–1.93)	−18.2	<0.001
ALT (U/L)	25 (13–86)	19 (14–27)	−10.0	<0.001
AST (U/L)	30 (19–82)	23 (19–27)	−16.3	<0.001
ALP (U/L)	122 (81–344)	78 (65–95)	−27.5	<0.001
GGT (U/L)	60 (21–366)	22 (15–35)	−23.7	<0.001
AFU (U/L)	17 (12–25)	20 (16–24)	−8.14	<0.001

Note: Mean ± SD for continuous variable and n (%) for categorical variable;

BMI was calculated by dividing weight (kg) by height squared (m^2^);

TC:Total cholesterol; HDL-C: high-density lipoprotein-cholesterol; LDL-C: low-density lipoprotein-cholesterol; ALT: alanine transaminase; AST: aspartate aminotransferase; ALP: alkaline phosphatase; GGT: gamma-glutamyl transpeptidase; AFU: alpha-L-fucosidase; FBG: fasting blood glucose

smoking and alcohol refer to ever smoking and ever alcohol drinking

Continuous variables are expressed as median values (interquartile range).

Supplementary table compared the sensitivity and specificity of tumor markers betwween PDAC patients and healthy controls. Statistically based sensitivity and specificity of CA199 was 74.2% and 95.9%, and CA199&CA242 was 62.8% and 100% in PDAC risk in the study. These detailed and specific data was available as reference indicator for PDAC diagnosis.

### Risk factors of case and control groups related to PDAC

In this analysis, major predictive indicators were significantly associated with increasing number of smoking, drinking, and TC/HDL-C ratio, family history of PDAC, chronic pancreatitis, cholelithiasis, prior history of cholecystectomy, and diabetes related to PDAC risk (P < 0.05, Table 2). Males that had a 1.68-fold risk in developing PDAC than females, implying that gender was associated with the risk of PDAC. There were statistically significant differences in the comparison of heavy smoking (>20 pack-year, OR = 2.4; 10~20 pack-year, OR = 2.2; <10 pack-year, OR = 1.0), heavy drinking (>1drink/day, OR = 2.2; 0~1drink/day, OR = 0.6), and new onset diabetes (<2 years, OR = 2.4; 2~5 years, OR = 1.7; >5 years, OR = 2.0) in the two groups, while the duration of CP (<8 years, OR = 34.9; >8 years, OR = 8.9) and cholecystectomy (<10 years, OR = 8.9; >10 years, OR = 6.0) did not play a primary role in statistic analyses.

**Table 2:** Univariate Analysis of Related Factors for PDAC.

**Variable**	**Cases**	**Controls**	**OR (95%C.I.)**	**P value**
	**N (%)**	**N (%)**		
Sex				
Female	469 (36.8)	1795 (49.4)	1.00 (referent)	
Male	805 (63.2)	1835 (50.6)	1.68 (1.47~1.91)	<0.001
Smoking (pack-year)				
None	745 (58.5)	2633 (72.5)	1.00 (referent)	
0~10	100 (7.8)	355 (9.8)	1.00 (0.77~1.26)	0.971
10~20	146 (11.5)	231 (6.4)	2.23 (1.79~2.79)	<0.001
>20	283 (22.2)	411 (11.3)	2.43 (2.05~2.89)	<0.001
Drinking (drink/day)				
None	835 (65.5)	2384 (65.7)	1.00 (referent)	
0~1	208 (16.3)	940 (25.9)	0.63 (0.53~0.75)	<0.001
>1	231 (18.1)	306 (8.4)	2.16 (1.79~2.60)	<0.001
Family History of PDAC				
No	1239 (97.3)	3582 (98.7)	1.00 (referent)	
Yes	35 (2.7)	48 (1.3)	2.11 (1.36~3.28)	0.001
Pancreatitis (year)				
None	1221 (95.8)	3624 (99.8)	1.00 (referent)	
0~8	47 (3.7)	4 (0.1)	34.9 (12.5~97.0)	<0.001
>8	6 (0.5)	2 (0.1)	8.9 (1.80~44.2)	0.007
Cholelithiasis				
No	1151 (90.3)	3323 (91.5)	1.00 (referent)	
Yes	123 (9.7)	307 (8.5)	1.16 (0.93~1.44)	0.194
Prior History of Cholecystectomy (year)				
None	1211(95.1)	3604 (99.3)	1.00 (referent)	
0~10	33 (2.6)	11 (0.3)	8.93 (4.50~17.7)	<0.001
>10	30 (2.4)	15 (0.4)	5.95 (3.19~11.1)	<0.001
Gallbladder polyps				
No	1246 (97.8)	3270 (90.1)	1.00 (referent)	
Yes	28 (2.2)	360 (9.9)	0.20 (0.14~0.30)	<0.001
Hypertension				
No	918 (72.1)	2326 (64.1)	1.00 (referent)	
Yes	356 (27.9)	1304 (35.9)	0.69 (0.60~0.80)	<0.001
Diabetes (years)				
none	1071 (84.1)	3333 (91.8)	1.00 (referent)	
<2	106 (8.3)	136 (3.7)	2.43 (1.86~3.16)	<0.001
2~5	36 (2.8)	65 (1.8)	1.72 (1.14~2.61)	0.01
>5	61 (4.8)	96 (2.6)	1.98 (1.42~2.75)	<0.001
TC/HDL-C ratio				
0~5	751 (72.6)	3397 (93.6)	1.00 (referent)	
>5	283 (27.4)	233 (6.4)	5.49 (4.54~6.65)	<0.001

Note: OR is adjusted for age and sex;

TC/HDL-C ratio: Total cholesterol/high-density lipoprotein-cholesterol ratio.

Related significant risk factors of PDAC were put through forward and backward stepwise regression (Table 3). After removing mixed factors, seven risk factors including heavy smoking, heavy drinking, FH of PDAC, chronic pancreatitis, diabetes, cholecystectomy, and high TC/HDL-C ratio were independently involved with the development of PDAC (Table 3), and the OR(95%CI) of each independent predictor was 2.11 (1.76~2.53), 1.83 (1.46~2.29), 2.79 (1.70~4.58), 28.9 (11.9~69.9), 2.04 (1.63~2.56), 8.01 (4.83~13.3), and 5.75 (4.71~7.03), respectively.

**Table 3:** Multivariate logistic regression analysis of risk factors for PDAC.

Variables	OR (95%C.I.)	P value
Heavy smoking	2.11 (1.76~2.53)	<0.001
Heavy drinking	1.83 (1.46~2.29)	<0.001
Family History of PDAC	2.79 (1.70~4.58)	<0.001
Chronic Pancreatitis	28.9 (11.9~69.9)	<0.001
Diabetes	2.04 (1.63~2.56)	<0.001
Prior History of Cholecystectomy	8.01 (4.83~13.3)	<0.001
TC/HDL-C ratio >5	5.75 (4.71~7.03)	<0.001

Note: Score of each variable is set to an integer of the logarithm of OR value;

OR: standardized for age and gender.

TC/HDL-C ratio: Total cholesterol/high-density lipoprotein-cholesterol ratio.

Heavy smoking: Smoking >10 pack-year; Heavy drinking: Drinking >1 drink/day.

### Risk score model in assessment on PDAC development

To calculate a risk score for estimating the risk of PDAC occurrence, the value of each independent predictor was taken to integer as one. We assigned all referents a value of 0, the score for participants was calculated by summing their total number of risk factors (Table 4). A predictive factor risk score consisting of 1 point each for the seven risk factors predicted an increased risk of PDAC. The total possible score was 7 points, and actual scores ranged from 0 to 4. Compared with the low score (0), the OR values at high scores were sharply increased after jointing two or more risk factors. The higher risk scores, the extremely higher PDAC risk. The calculation of p for trend was proven to be statistically significant for the trend test.

**Table 4:** Scoring System of PDAC Risk Pattern in Each Score Category.

Risk score^*^	Total Number	Controls (N = 3630)	Cases (N = 1034)			
			N	%	OR (95% CI)	p-value
0	2729	2403	326	11.9	1.00 (referent)	
1	1300	930	370	28.5	3.06 (2.57~3.65)	<0.001
2	497	263	234	47.1	7.08 (5.63~8.91)	<0.001
3	107	28	79	73.8	22.4 (14.2~35.4)	<0.001
4	31	6	25	80.6	31.4 (12.7~77.5)	<0.001
P_trend_						<0.001

Note: *Range for score was 0~7 actual points for 7 independent risk factors of PDAC, comprising heavy smoking (no, 0 points; yes, 1 point), alcohol consumption more than 1 drink/day (no, 0 points; yes, 1 point), family history of PDAC (no, 0 points; yes, 1 point), chronic pancreatitis (no, 0 points; yes, 2 points), cholecystectomy (no, 0 points; yes, 1 point), diabetes (no, 0 points; yes, 1 point), TC/HDL-C ratio (0~5, 0 points; >5, 1 point).

P for trend was 2-sided and based on the integer score for exposure risk of each level.

OR: adjusted for age and sex. Missing data was 240 due to the lacking blood lipid profile in the case group.

## Discussion

Without particularly strong risk factors or early detection tests, the PDAC patients suffered an extremely poor 5-year survival rate. Curative resection offers the only chance for long-term survival, which depends on early diagnosis of PDAC patients. Many researches on predisposing risk factors for the development of PDAC have been carried out for decades.

At univariate analysis, smoking is currently the only internationally recognized behavioral risk factor of PDAC, and 25% of PDAC result from smoking^9^. Large amount of cigarette consumption contributed to 2.11 times increase in PDAC compared to non-smokers, and the risk estimates increased with the increasing amount of cigarettes. Smoking was associated with PDAC by 2~3 fold risks in many authoritative literatures^5^. which may have a late-stage effect on pancreatic carcinogenesis even 15 years after smoking cessation^24^. Although pancreas is not directly exposed to tobacco like lung, carcinogens in tobacco can be indirectly absorbed by lung through bloodstream into pancreas or by directly absorbed through digestive tract to the duodenal that refluxes to pancreas, causing detrimental effects^10^. If the second mechanism exists, it can be explained to a certain extent why the majority of PDAC took place in the head of pancreas.

Alcohol consumption on the risk of PDAC remains controversial^11^, and there was an increased risk of PDAC among male heavy alcohol drinkers in a pooled analysis^12^. Alcohol is not only increase the reaction of pancreas to cholinergic and pancreozymin, but it also can increase the brittleness of pancreatic lysosomal enzymes and the activity of trypsin, making gradual destruction of pancreatic parenchymathe and occurrence of PDAC. The toxicity of acetaldehyde, upregulation of inflammatory and immunosuppressive reactions may also play a role in pancreas carcinogenesis^12^.

Preexisting and new-onset DM both have been suggested a strong risk factor of PDAC in available studies^5,15^. In this study, vast majority DM of case group was newly detected. Isaksson^16^ demonstrated that defects in insulin activity and glucose transport contributed to pancreatic cancer-associated insulin resistance on cellular level. Increasing evidences suggested that insulin resistance and subsequent hyperinsulinemia are common phenomenon in diabetes^17^, liver enzymes abnormalities^29^ and dyslipidemia^30^, which have been verified associated with the promotion of PDAC development *in vivo* and vitro^17,18^.

Nevertheless, a paucity of epidemiologic studies had examined the correlation of gallbladder disease, cholecystectomy^31–34^, and dyslipidemia^30,35,36^ to the risk of PDAC development. Cholecystectomy had been proposed as potential risk factors for the development of cancers of ampulla and pancreas^32,33^. An estimated 9~l5% adults are afflicted with gallstones worldwide^37^. Meanwhile, over 0.7 million cholecystectomies are performed in the United States every year^38^. Cholecystectomy was reported to enhance pancreatic tumor formation in experimental animals^34^. The presumed increased release of cholecystokinin was probably an culprit for the increased long-term risk of PDAC following cholecystectomy^32^.

We found that the ratio of TC/HDL-C independently associated to the risk of PDAC. Chen’s analysis suggests that a high intake of cholesterol could increase the risk of pancreatic cancer, especially in American society^39^. We also discover at early time of the correlation between cholecystectomy and PDAC, which fit with Lin^32^ that found a 23% risk related to cholecystectomy.

A history of CP has been widely considered as a risk factor for PDAC^5^. The duration of CP was suggested to be correlate with the degree of K-ras gene mutations^20^. We had excluded patients who got cholelithiasis with CP to cancel the possibility that CP may confound the association between cholelithiasis and PDAC.

In this study, obesity does not show a clear association with the occurrence of PDAC. Becides, Chinese body type is usually slim, and the majority of PDAC patients before the Whipple operation are thin in the ward.

The highlights in the current study confirm those previously identified individual single factors into one overall model of an integer-based risk score system for evaluate setting on cumulative risk of PDAC. A highly statistically significant association of the risk score with all seven outcome measures was evaluated. Few institutions reported the selection algorithm of predictive risk score model to evaluate cancer risk^40,41^. Previously proposed models, such as the APACHE or the POSSUM scoring system, are usually applicable in estimate of postoperative severity^42^. However, they are difficult to be calculated and not suitable for preoperative probability of illness. In this population-based analysis, we have shown an effective demonstration of the joint effects of combined risk factors for PDAC, which provide a potential means to stratify individual risk at early stages. In a American hospital, a total of 1,616 subjects (808 PDAC patients and 808 healthy controls) were enrolled in a case-control study. It is found that smoking, family history, heavy alcohol consumption, diabetes mellitus, and history of pancreatitis were significant risk factors for pancreatic cancer, which is almost same to our study^31^. Our study newly discovered two risk factors: prior history of cholecystectomy and TC/HDL-C ratio (+).

We found that combination of risk factors, in scoring system, had tighter relationships with the development of PDAC than that of a single risk factor. When two or more risk factors were considered into combination, strong association contributed to the development of PDAC. Those single risk factors related to PDAC were *a priori* expected. Our data indicate that it would be better to predict and prevent the PDAC development by using multifactorial analysis.

The combined risk factors may trigger a possibly significant synergistic effect, which may amplify PDAC risk. It was believed that the combined risk factors could stimulate the growth of PDAC cells via energy balance^43^. The plausible effects of each factor are pleiotropic in nature with likely overlapping influence on noted pathways thought to be relevant to PDAC development, including lifestyle effects, insulin resistance, metabolic influence, and so on. For example, Ben^11^ and Dite^44^ advocated a synergistic effect of smoking and diabetes on PDAC risk. It was speculated that smoking combined with chronic pancreatitis was associated with a rather high risk of PDAC. Talamini^45^ proposed N-nitroso compounds that originated from tobacco may play an important role on the pancreatic ductal active cells that drew from chronic pancreatitis patients, thereby increasing the risk of PDAC. In populations of chronic pancreatitis, the life-time risk of PDAC development is nearly 40% with high rates of smoking, while could below 20% for non-smokers^46^. Patients could suffer an increased susceptibility to pancreatic DNA damage, chronic inflammation and becoming cancerous by smoking-induced oxidative stress^31^. It has been reported that bile acids or certain metabolites in the bile may have a carcinogenic effect, and bile might contain chemical carcinogens that derived from tobacco, in case that someone is a current smoker^32^. Patients with diabetes and a history of CP had a 12-fold risk of PDAC than patients with either condition alone in Brodovicz’s studies^47^. It implied that alcoholic pancreatitis has a positive association in risk of PDAC^48^. About 70% of pancreatitis cases are believed to be attributable to heavy alcohol consumption^49^. In addition, if high-fat diet and smoking coexist, cholecystokinin and carbachol that associated with lipid metabolism will stimulate the accumulation of nicotine in pancreatic acini, and synergy of the two may even induce PDAC^50^. In addition, a pooled analysis included a big number of PDAC cases concluded that heavy drinkers (>3 drinks/day) can result in an increased risk of PDAC^51^. Genkinger^12^ and Michelle^13^ with their teams demonstrated a modest increase in risk of PDAC by heavy amounts of alcohol use but not by the type of alcohol. Tong^52^ and colleagues suggested that PDAC risk decreased as duration increased since diagnosis of pancreatitis. Our results are consistent with these previous reports.

This study provided sufficient evidence and showed clues for the risk prediction of PDAC by virtue of observation for various kinds of data between PDAC patients and health controls. Understanding pathways of combined risk for PDAC may provide perspectives into pancreatic carcinogenesis. If the above research is confirmed, a better understanding of the etiology and an earlier detection of PDAC may help to reduce its incidence. The risk score for PDAC described here can serve as a model from which other studies may develop similar systems.

Of note, the major advantages are represented as follows: First, large number and high quality for the accuracy of the diagnostic confirmation of the patients group, restriction of microscopically confirmed cases generating the most valid estimates of risk. Second, adequate size and high representative of the control group. Third, large-scale collection and the use of multiple indicators of PDAC risk factors. Fourth, we minimized selection bias by including all cases and matched controls within the selected time period, adjusting them through appropriate and rigorous statistical methods. However, there are some limitations to the present study. First, due to a case-control study, it is unable to implement years of following-up direct exposure in developing PDAC. Second, a restrospective study is susceptible to have potential bias and selection bias. Third, because of the long age, some patients’ lipid profiles are lost, but this did not affect the overall outcome.

In summary, we have designed a risk score system to evaluate the risk of PDAC in high-risk populations. The results shown provided the best demonstration of the joint effects of combined risk factors for PDAC. This prediction risk score incorporating seven risk factors should be used for promotion and popularization. We believe that such a tool may be helpful in conjunction with early diagnosis of pancreatic cancer and allow for an accurate comparison of cancer predictors between institutions. It needs further validation using prospective evaluation in future clinical trials.

## Electronic supplementary material

Supplemental Material
